# Identification of a Chlorovirus PBCV-1 Protein Involved in Degrading the Host Cell Wall during Virus Infection

**DOI:** 10.3390/v13050782

**Published:** 2021-04-28

**Authors:** Irina V. Agarkova, Leslie C. Lane, David D. Dunigan, Cristian F. Quispe, Garry A. Duncan, Elad Milrot, Abraham Minsky, Ahmed Esmael, Jayadri S. Ghosh, James L. Van Etten

**Affiliations:** 1 Nebraska Center for Virology, University of Nebraska-Lincoln, Lincoln, NE 68583-0900, USA; ddunigan2@unl.edu (D.D.D.); quispecristian@gmail.com (C.F.Q.); gduncan@nebrwesleyan.edu (G.A.D.); jghosh2@unl.edu (J.S.G.); 2 Department of Plant Pathology, University of Nebraska-Lincoln, Lincoln, NE 68583-0722, USA; lclane2@gmail.com; 3 Department of Structural Biology, The Weizmann Institute of Science, Rehovot 76100, Israel; eladm@iibr.gov.il (E.M.); avi.minsky@weizmann.ac.il (A.M.); 4 Botany and Microbiology Department, Faculty of Science, Benha University, Qalubiya Governorate, Banha 13511, Egypt; a7medesmael@gmail.com

**Keywords:** Chlorella, *Phycodnaviridae*, chlorovirus, PBCV-1, algal lytic activity, cell wall degrading activity, alginate-like lyase

## Abstract

Chloroviruses are unusual among viruses infecting eukaryotic organisms in that they must, like bacteriophages, penetrate a rigid cell wall to initiate infection. *Chlorovirus* PBCV-1 infects its host, *Chlorella variabilis* NC64A by specifically binding to and degrading the cell wall of the host at the point of contact by a virus-packaged enzyme(s). However, PBCV-1 does not use any of the five previously characterized virus-encoded polysaccharide degrading enzymes to digest the *Chlorella* host cell wall during virus entry because none of the enzymes are packaged in the virion. A search for another PBCV-1-encoded and virion-associated protein identified protein A561L. The fourth domain of A561L is a 242 amino acid C-terminal domain, named A561L^D4^, with cell wall degrading activity. An A561L^D4^ homolog was present in all 52 genomically sequenced chloroviruses, infecting four different algal hosts. A561L^D4^ degraded the cell walls of all four chlorovirus hosts, as well as several non-host *Chlorella* spp. Thus, A561L^D4^ was not cell-type specific. Finally, we discovered that exposure of highly purified PBCV-1 virions to A561L^D4^ increased the specific infectivity of PBCV-1 from about 25–30% of the particles forming plaques to almost 50%. We attribute this increase to removal of residual host receptor that attached to newly replicated viruses in the cell lysates.

## 1. Introduction

A major difference between viruses that infect bacteria and eukaryotic organisms is the initial events associated with infection. Typically, bacteriophages are confronted with the problem of binding to and penetrating a bacterial cell wall (the wall is often referred to as the outer membrane or outer cell envelope) in order to eject their genome into the interior of the cell. Penetration of the bacterial wall typically involves a virus-packaged wall digesting enzyme(s), often referred to as a peptidoglycan hydrolase or endolysin [1], frequently located in a tail structure of tailed phages. The ejection of phage DNA into the cell usually results in an empty virus capsid remaining on the outside of the cell. In contrast to bacteriophages, viruses that infect animal cells only have to deal with a plasma membrane, and after attachment to the membrane, typically the entire nucleocapsid particle is taken into the cell either by endocytosis or phagocytosis, or by membrane fusion [2]. Therefore, un-coating of the viral genome typically occurs inside the host cell or at the plasma membrane. Viruses that infect higher plants also have to deal with cell walls but they avoid the issue by either infecting wounded cells (mechanical infection) or a biological vector transfers the particles to the inside of the plant cell and the viruses subsequently move cell-to-cell via plasmodesmata.

The large icosahedral, plaque forming, dsDNA chloroviruses (family *Phycodnaviridae*, genus *Chlorovirus*) are unusual among viruses infecting eukaryotic organisms in that they, like bacteriophages, need to penetrate a rigid algal cell wall to initiate infection. They infect freshwater, unicellular, eukaryotic green algae, which normally exist as mutualistic endosymbionts in protists and are often referred to as zoochlorellae. Chloroviruses fall into four clades based on their hosts [3]. NC64A viruses infect *Chlorella variabilis* NC64A [4,5], Osy viruses infect *Chlorella variabilis* Syngen 2-3 [6], SAG viruses infect *Chlorella heliozoae* [4] and Pbi viruses infect *Micractinium conductrix* [7].

The prototype *Chlorovirus* Paramecium bursaria chlorella virus (PBCV-1) is an icosahedron (190 nm in diameter) with a spike structure at one vertex [8], which appears to make the first contact with the cell wall of its host, *C. variabilis* NC64A [9]. Attachment is immediately followed by cell wall degradation precisely at the point of contact by a virus-packaged enzyme(s) [10]. Following wall degradation the viral internal membrane fuses with the host membrane [11] causing immediate host membrane depolarization [12] and potassium ion efflux [13]. These events lower the turgor pressure of the host and facilitate entry of the viral DNA and virion-associated proteins into the cell [14], leaving an empty viral capsid on the cell surface [10]. Therefore, the initial infection process or un-coating of the genome is similar to that of many bacteriophages.

Degradation of the *C. variabilis* NC64A wall is associated with two events in the PBCV-1 life cycle. Besides entry into the cell, nascent infectious viruses exit the cells at 6 to 8 h post infection (PI) by cell lysis [15]. It is not known if chloroviruses use the same set of enzymes for both events. The 331 kb linear PBCV-1 genome has 416 putative protein coding sequences (CDSs) [16]. About 50% of these CDSs resemble known proteins including two chitinases [17,18], a chitosanase [18,19], a β-1,3 gluconase [20], and a polysaccharide lyase, cleaving chains of β- or α-1,4-linked glucouronic acids [21,22], hereafter referred to as vAL-1. Recombinant proteins have been produced from each of these five genes and shown to have the predicted polysaccharide degrading enzyme activity [23].

Furthermore, previous experiments indicate that a crude enzyme preparation made from PBCV-1 lysates, named wLysin (wall Lysin as compared to virion isolated, vLysin), has good wall degrading activity and can lead to *C. variabilis* NC64A protoplast formation [24,25]. Therefore, it was assumed that one or more of these five PBCV-1-encoded enzymes were packaged in the PBCV-1 virion and were responsible for degrading the host cell wall at the point of infection. In fact, a chitosanase was reported to be packaged in the virion of a closely related chlorovirus, CVK2 [19]. However, a subsequent report [17] indicated that the chitosanase activity associated with the CVK2 particles was due to incomplete purification of the virion. Subsequently, a PBCV-1 proteomic study of highly purified virions identified 148 virus-encoded proteins and one host encoded protein [16]. Unexpectedly, however, none of the five polysaccharide-degrading enzymes are packaged in the PBCV-1 virion.

In this report, we have re-examined the 148 virus-encoded proteins that are packaged in the PBCV-1 virions for possible polysaccharide or cell wall degrading activity. The result was that a putative polysaccharide hydrolase domain was identified in one of the PBCV-1 encoded proteins packaged in the virion, CDS A561L (GenBank https://www.ncbi.nlm.nih.gov/protein/9632119, gene ID: 917870, accession no. NP_048917). Therefore, we tested the hypothesis that A561L is involved in the digestion of the cell wall at the point of viral attachment.

## 2. Materials and Methods

### 2.1. Viruses and Host Strains

The virus PBCV-1 host *C. variabilis* NC64A was grown in modified Bold’s basal medium (MBBM) [26]. All other green algae were also grown in MBBM with some exceptions: *C. variabilis* NIES 2540 and *C. variabilis* NIES 2541 were grown in MBBM supplemented with 1mL/L 1% thiamin; *M. conductrix* Pbi was grown in FES medium [27]. PBCV-1 virus production, ultra-purification and plaque assay of viruses was carried out as described previously [26,28,29].

### 2.2. Recombinant A561L and A561L^D4^

For protein overexpression the Champion pET Directional TOPO cloning kit was employed (Thermofisher Scientific, Waltham, MA, USA). PBCV-1 open reading frame *A561L* and truncated *A561L^D4^* encoding the 247 amino acid C-terminal region of A561L were cloned from PCR-amplified viral DNA. The primers were designed to generate compatible overhangs following the pET 102/DTOPO plasmid kit guidelines (Thermofisher Scientific, Waltham, MA, USA). Cloned DNA fragments were verified by sequencing. Plasmids were multiplied using One Shot^®^ TOP10 chemically competent *E. coli* and were conserved at −80 °C in 20% glycerol stocks.

Recombinant proteins were expressed in BL21 Star (DE3) *E. coli*, using 0.5 mM IPTG following the pET102/DTOPO kit guidelines and the Ni-NTA agarose affinity chromatography matrix for purifying recombinant proteins carrying a His tag. The soluble protein size was approximately 42 kDa with the His-Patch thioredoxin tag (provides an efficient fusion partner for translation of the fusion protein), V5 epitope tag (for detection of the fusion protein by the anti-V5 antibodies) and 6xHis tag.

### 2.3. vLysin Preparation and Activity Assay

A PBCV-1 virus preparation (4 mg/mL) in TB, (50 mM Tris HCl, pH 7.8) was mixed with an equal volume of lysing solution [50 mM KOH, 100 mM MOPS (pH 7.0), 10 M LiCl, 4 mM Na_2_EGTA], incubated for 50 min at 37 °C then spun for 20 min at 21,000× *g* and the supernatant fraction was collected. The supernatant fraction containing vLysin, was dialyzed for 24 h at 4 °C against 3 changes of 25 mM KOH, 50 mM MOPS (pH 7.0).

### 2.4. Cell Wall Degrading Activity Assayed by Chlorophyll Release

The assay measured chlorophyll release as a result of cell wall digestion of the algae [30]. Chlorella cells were freshly harvested from 4-day-old cultures (1.0–1.5 × 10^7^ cells/mL) by centrifugation at 4000× *g* for 3 min, washed one time with sterile TB and then re-suspended in TB at a concentration of 2.5 × 10^7^ cells/mL. Unless otherwise indicated, lysis activity was determined in 100-µL reaction mixtures in TB supplemented with 10 mM CaCl_2_ using *C. variabilis* NC64A cells at a concentration of 2.5 × 10^7^/mL with specified amounts of A561L^D4^ followed by incubation for 1 h at room temperature. To facilitate chlorella cell wall rupture by the protein, SDS was added to 1% concentration (final) and incubated for 1 min at room temperature, then centrifuged for 15 min at 16,000× *g*. The optical densities of the supernatant fractions were determined in a Beckman DU 530 UV/Vis spectrophotometer (Beckman-Coulter, Indianapolis, IN, USA) at a λ of 420 nm. The amount of detected chlorophyll served as an indicator of the extent of cell wall degradation by the recombinant protein.

The determination of optimal temperature for A561L^D4^ was performed at 10 °C, 15 °C, 20 °C, 25 °C, 30 °C, 37 °C, 45 °C, 50 °C, 55 °C and 60 °C with 10 μg/mL A561L^D4^ concentration. To evaluate an optimal pH for lysing activity an array of 100 mM Tris-citrate buffers ranging in pH from 2.4 to 9 was used. Divalent-cation experiments were performed in the same reaction mixture by replacing CaCl_2_, with 10 mM MgCl_2_, MnCl_2_, CuCl_2_, CoCl_2_, FeSO_4_, ZnSO_4_, NiCl_2_, EDTA or EGTA.

### 2.5. Protein Activity Assayed by Calcofluor-White (CFW) Release

The preparation of *C. variabilis* NC64A ghost cells has been described elsewhere [25]. Briefly chlorella cells at a concentration of 1–1.5 × 10^7^ cells/mL, were harvested and extracted multiple times with methanol until the chlorophyll was removed. To remove traces of methanol, the ghost cells were washed three times with TB before an experiment. Chlorella ghost cells at 5 × 10^7^ were stained with 0.001% (final concentration) CFW for 30 min, then washed 4 times with TB and re-suspended in TB. Cell wall degrading activity was determined in a 100 µL reaction mixture in TB containing 4 × 10^6^ (total) chlorella ghost cells and the indicated amount of protein. After 1 h incubation at room temperature the reaction mixture was spun at 16,000× *g* for 15 min, the supernatant fraction was removed and released CFW was measured in a Beckman DU 530 UV/Vis spectrophotometer at λ = 215 nm.

### 2.6. Effect of A561L^D4^ Protein Treatment of C. variabilis Ghost Cells on PBCV-1 Virus Attachment

Ghost cells (10^8^ cells/mL) in TB with 10 mM CaCl_2_ were treated with various amounts of A561L^D4^ at room temperature. After 1 h of incubation of ghost cells, the reaction was spun for 30 s at 16,000× *g*, the supernatant fraction was collected for future experiments and pelleted ghost cells were washed with TB one time, then re-suspended in 200 µL of TB with 10 mM CaCl_2_.

PBCV-1 virus was added to the treated ghost cells at a MOI of 10 and incubated at room temperature. The control was virus incubated in TB with 10 mM CaCl_2_. After 1 h incubation, serial dilutions of the reactions were made and virus infectivity was plaque assayed.

### 2.7. Effect of Soluble Products (Possibly Receptors) Resulting from A561L^D4^ Treatment of C. variabilis NC64A Ghost Cells on PBCV-1 Virus Infectivity

NC64A ghost cells (10^8^ cells/mL) in TB with 10 mM CaCl_2_ were treated with different amounts of recombinant A561L^D4^. After incubation for 1 h at room temperature, treated ghost cells were spun for 15 min at 16,000× *g* and the supernatant fractions were collected. PBCV-1 virus was added to the collected supernatant fractions at a concentration of 10^9^ PFU/mL and incubated for 1 h. Serial dilutions of the reactions were made and virus infectivity was plaque assayed.

### 2.8. Electron Microscopy

*C. variabilis* NC64A cells (5–6 × 10^7^ cells/mL) were treated at room temperature with 250 µg/mL recombinant A561L^D4^ protein for 10 min and then concentrated by centrifugation. The electron microscopy studies of the cell wall digesting protein were carried out as described elsewhere [31]. Briefly, NC64A cells were fixed with 2% of glutaraldehyde for 2 h at room temperature. Then, cells were centrifuged and resulted pellets were embedded in 3.4% agar (Difco). The pellets were trimmed and stained with 1% *v/v* of osmium tetra-oxide in 0.1 M cacodylate buffer at room temperature. Then, samples were washed with distilled deionized water and stained with 2% water solution of uranyl acetate. Cells were dehydrated in graded ethanol concentrations, and subsequently embedded in Eppon. Thin sections (70–100 nm) were cut by Ultracut UCT microtome (Leica) and post stained with 2% uranyl acetate and Reynold’s lead citrate. Images were obtained using FEI Spirit TEM operated at 120kV and recorded on a CCD camera (Eindhoven, the Netherlands).

### 2.9. SDS-PAGE and Western Blots

PBCV-1 virion proteins were solubilized and separated by sodium dodecyl sulfate-polyacrylamide gel electrophoresis (SDS PAGE) as described elsewhere [32]. Resolved protein gels were either stained with Coomassie brilliant blue R-250 or transferred to a nitrocellulose membrane and hybridized with PBCV-1 A561L^D4^ antiserum produced in mice (dilution 1:500). Goat produced anti-mouse IgG conjugated to horseradish peroxidase (ThermoFisher Scientific, Waltham, MA, USA) was used as the secondary antibody (dilution 1:1000). SuperSignal West Pico PLUS Chemiluminescent Substrate was used for visualization (ThermoFisher Scientific, Waltham, MA, USA).

### 2.10. Effect of Anti A561L^D4^ Antibody on vLysin Activity

PBCV-1 vLysin isolated from ultra-purified virions was treated with anti A561L^D4^ antibody (AB) produced in mice (800-fold dilution). After 1 h incubation, to remove vLysin neutralized with anti-A561L^D4^ antibodies from the reaction, magnetic beads (MB) coupled with anti-mouse Ig (RayBiotech, GA, USA, cat # 801–103) were added to the reaction and the supernatant fraction was separated from the MB following the manufacturer’s instructions. The resultant supernatant fraction that presumably had vLysin activity neutralized and removed was mixed with NC64A cells at concentration 6 × 10^7^ cells/mL and incubated for 1 h. SDS was added to the treated cells to 1% concentration (final) and incubated for 1 min at room temperature, then NC64A cells were centrifuged for 15 min at 16,000× *g*, the supernatant fraction was removed and chlorophyll release was measured using a spectrophotometer (Beckman DU 530) at λ = 420. Untreated PBCV-1 vLysin was used as a positive control. NC64A cells treated with pre-bleed serum and 1% SDS served as a negative control.

### 2.11. Phylogenetic Analyses

A summary of the 52 homologs of A561L^D4^ protein analyzed in this study are provided in Supplementary Table S1. Based on these sequences multiple-sequence alignments were created using the ClustalW alignment with the Geneious 11.0.5 program (Biomatters Ltd., Auckland, New Zealand, https://www.geneious.com, accessed on 27 April 2021), and the phylogenetic tree was constructed with Geneious plugin program PhyML 3.3.20180621 (Maximum likelihood) [33] using the default settings. To identify domains of the protein A561L, encoded by gene *a561l*, fragments of different lengths were used as queries in BLASTp searches [34] against a data base of proteins in UniProt [35] with scoring matrix BLOSUM62.

A 3D structure model of A561L^D4^ domain was constructed using normal mode of the Phyre2 Server (Protein Homology/analogY Recognition Engine V 2.0) [36]. Phyre2 results were visualized and edited using VMD molecular graphics viewer software [37].

## 3. Results and Discussion

### 3.1. PBCV-1 A561L Is a Candidate for Degrading the Host Cell Wall

As noted in the introduction, PBCV-1 encodes five enzymes [23] that either alone or in combination were candidates for the presumed enzyme activity that degrades the host cell wall during the initial phase of virus infection. However, none of these proteins were detected in the virion proteome study [16].

**Figure 1:**
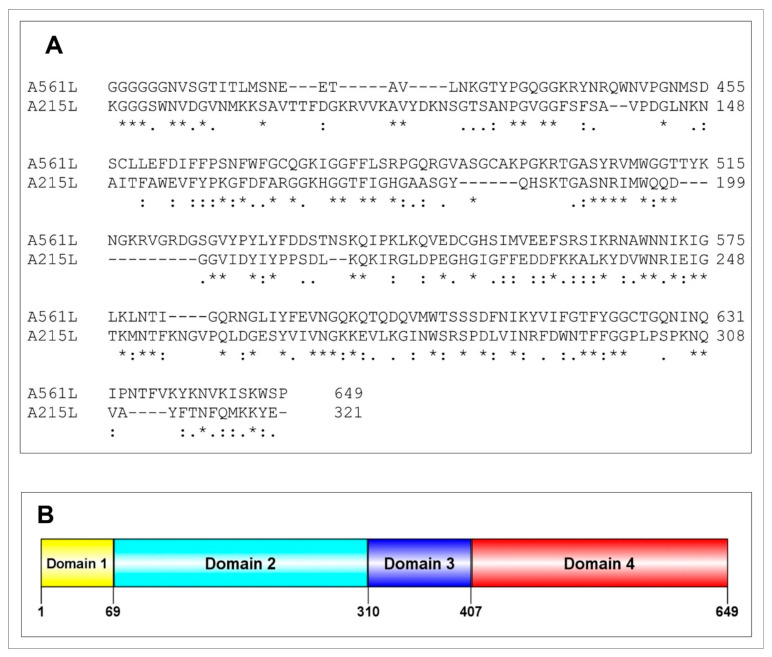
A561L protein characteristics: domains and possible functions: (**A**) Amino acid sequence pairwise alignment of a carboxyl-terminal domain of CDS A561L with the carboxyl terminus of CDS A215L; A215L was previously characterized as a polysaccharide lyase [vAL-1 protein] [38]. (**B**) Predicted domains in A561L—domain 4 is the part of the protein that resembles A215L (domains were identified using InterPro domain prediction database [39] with manual curation). Domain 1 has a transmembrane domain and the closest match is a transporter protein encoded by *Listeria kieliensis* although the E-value was low, 3.6 × 10^−1^; Domain 2 has the best hit to a TolA protein of *Trichomonas vaginalis* (Tol proteins are involved in the stability of the outer membrane in Gram-negative bacteria) with an E-value of 4.1 × 10^−14^ but it also resembles a peptidase S8 domain-containing protein from *Tetradesmus obliquus* with an E-value of 9.3 × 10^−8^; Domain 3 resembles a LEA 2 domain-containing protein (late embryogenesis abundant protein, which plays a role in abiotic stress response and tolerance in plants and are part of the hypersensitive response to plant pathogen infection) encoded by *Selaginella moellendorffii* with an E-value of 6.9 × 10^−6^; and Domain 4 resembles an alkaline alginate lyase/1–4 polyglucuronic acid lyase/polysaccharide lyase family 14 with an E-value of 2.1 × 10^−14^.

Therefore, another bioinformatics search of the 148 PBCV-1-encoded, virus-packaged CDSs was conducted to determine if any of them might be candidates for degrading the host cell wall. The 649 amino acid A561L CDS became a candidate because its C-terminal 242 amino acid domain has 32% and 50% amino acid identity and similarity, respectively, over 211 amino acid residues with the C-terminal portion of CDS A215L (Figure 1A). A215L is one of the previously characterized PBCV-1 polysaccharide degrading enzymes, referred to as vAL-1 [38].

**Figure A1:**
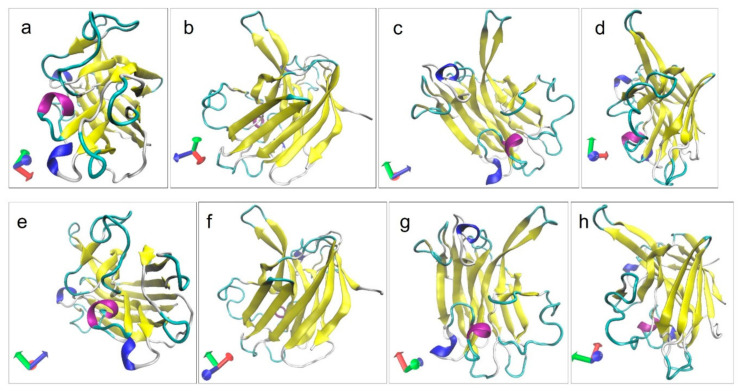
Predicted alginate lyase structure of A561L^D4^ protein based on remote homology. Protein ribbon model colors are based on a secondary structure. Top row (**a**–**d**): views of A561LD4 3D protein model from different dimensions; bottom row (**e**–**h**): view of 3D crystal structure of the marine pl-14 alginate lyase from *Aplysia kurodai* from different dimensions.

In silico protein structure model of A561L^D4^ protein by Phyre2 analysis identified alginate lyase based on 86.8% coverage with 100% confidence (Table A1) from multiple available protein structures derived from the protein data bank (PDB). A crystal structure of the marine PL-14 alginate lyase from *Aplysia kurodai* served as a model (Figure A1 of Appendix A).

**Figure A2:**
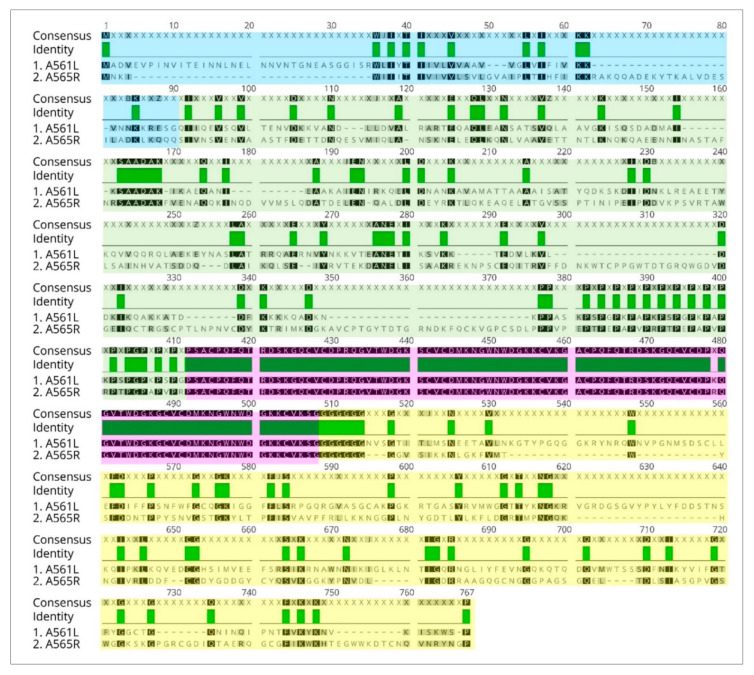
Pairwise amino acid alignment of A561L and A565R PBCV-1 proteins using Geneious 11.0.5 software. Green color denotes identical amino acids. Predicted domains in A561L: Domain 1 is highlighted in blue (69 aa); Domain 2 is highlighted in green (241 aa); Domain 3 is highlighted in pink (96 aa); and Domain 4 highlighted in yellow is an alginate lyase domain (242 aa).

A561L has three additional domains (Figure 1B): (i) a 69 amino acid domain 1 is a transmembrane domain and has some resemblance to a transporter protein, (ii) a 241 amino acid domain 2 resembles a TolA protein and (iii) a 97 amino acid domain 3 is similar to a LEA 2 domain-containing protein (late embryogenesis abundant protein) that is nearly identical to the PBCV-1 A565R CDS (Figure A2 of Appendix A). Thus, the A561L CDS became the focus of this study.

### 3.2. Development of Assays for Chlorella Cell Wall Degrading Activity

**Figure 2:**
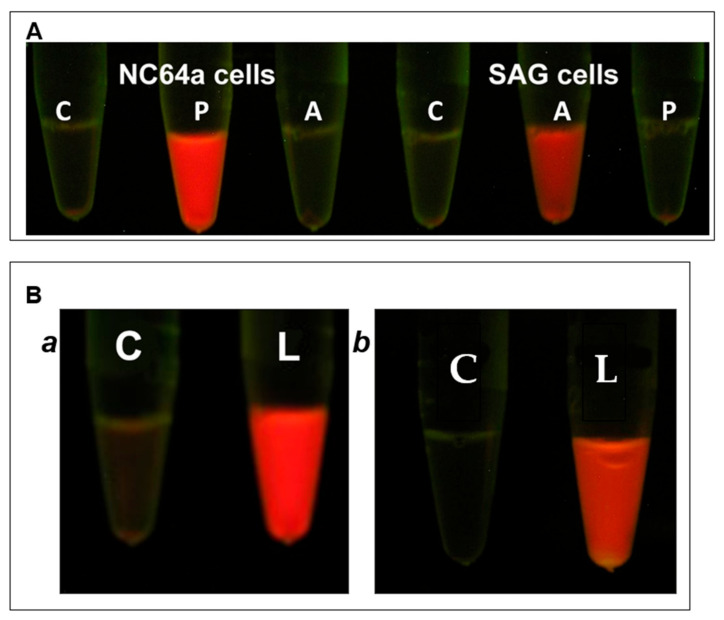
Specificity of the reaction of chlorella cells to virus infection and to virion associated cell wall degrading activity. (**A**). Exposure of chlorella host cells to chloroviruses PBCV-1 and ATCV-1. From left to right: (C) control (intact *C. variabilis* NC64A cells treated with 1% SDS), (P) *C. variabilis* NC64A cells plus intact PBCV-1, (A) *C. variabilis* NC64A cells plus intact ATCV-1 virus, (C) control (intact *C. heliozoae* SAG cells treated with 1% SDS), (A) *C. heliozoae* SAG cells plus intact ATCV-1 virus, (P) *C. heliozoae* SAG cells plus intact PBCV-1 virus. Cells (concentration 2 × 10^7^) were infected with viruses at a MOI of 5 incubated for 15 min and SDS was added to 1%. (Note: One obtains the same results if one uses Triton X-100 at the same concentration.) Note the specificity of chlorovirus infection. (**B**). Exposure of chlorella host cells to PBCV-1 vLysin: Panel ***a***: *C. variabilis* NC64A cells, Panel ***b***: *C. heliozoae* SAG cells. (C) Control (intact cells treated with 1% SDS), (L) cells incubated with PBCV-1 vLysin. Cells (concentration 2 × 10^7^) were treated with activity equivalent to 5 × 10^10^ PFU/mL for 15 min followed by addition of SDS. Note that the PBCV-1 vLysin treatment resulted in the release of chlorophyll from both types of cells. Images were taken under UV light to evaluate the autofluorescence of chlorophyll (red).

As described in the Materials and Methods section, two assays were developed for qualitative and quantitative assessment of chlorella cell wall degrading activity of A561L^D4^. The first is a quick and simple qualitative assay that relies on the natural resistance of the chlorella cells to nonionic and anionic detergents. To illustrate the assay, chlorovirus PBCV-1 particles were added to *C. variabilis* NC64A cells at a multiplicity of infection (MOI) of 5 and incubated for 15 min; 1% sodium dodecyl sulfate (SDS) was then added to the cells, the cells were centrifuged in a microcentrifuge and chlorophyll fluorescence was monitored in the supernatant fraction. Note that the virus infected cells led to the release of chlorophyll (red fluorescence color), whereas no chlorophyll was released from the control cells (Figure 2A). Similar results were obtained with C. heliozoae cells infected with virus ATCV-1 (Figure 2A) and M. conductrix cells infected with virus CVM-1 (results not shown). Addition of the viruses to non-host cells did not result in chlorophyll release because the viruses are specific for their hosts and do not attach to non-host cells [3].

Experiments to extract a cell wall degrading enzyme(s) from the three purified viruses led to the discovery that exposure of the viruses to 5 M LiCl, followed by centrifugation, released a soluble fraction (termed vLysin) that made the three algal cells susceptible to 1% SDS. Furthermore, exposure of the three virus hosts to the vLysin from each of the viruses revealed that all the cells were susceptible to 1% SDS. Therefore, the cell wall degrading activity packaged in the virions was not as specific as the viruses, i.e., the activity had a broader cell-type range than the viruses (Figure 2B).

The second assay employed chlorella ghost cells (algal cells extracted with methanol) dyed with calcofluor-white (CFW) [25]. Cell wall degrading activity was monitored by the amount of released dye.

### 3.3. Expression and Purification of A561L^D4^

To explore A561L potential cell wall degrading activity two recombinant proteins were produced—intact A561L and a truncated portion of A561L that only contained the vAL-1 like domain, amino acids 413 to 649 (referred to as A561L^D4^). Despite trying many procedures, the intact recombinant A561L protein remained insoluble. However, most of the A561L^D4^ was in the soluble phase and the His-tag expressed protein was purified over a Ni-binding column. One liter of *E. coli* culture produced about 10 mg of soluble recombinant A561L^D4^.

**Figure 3:**
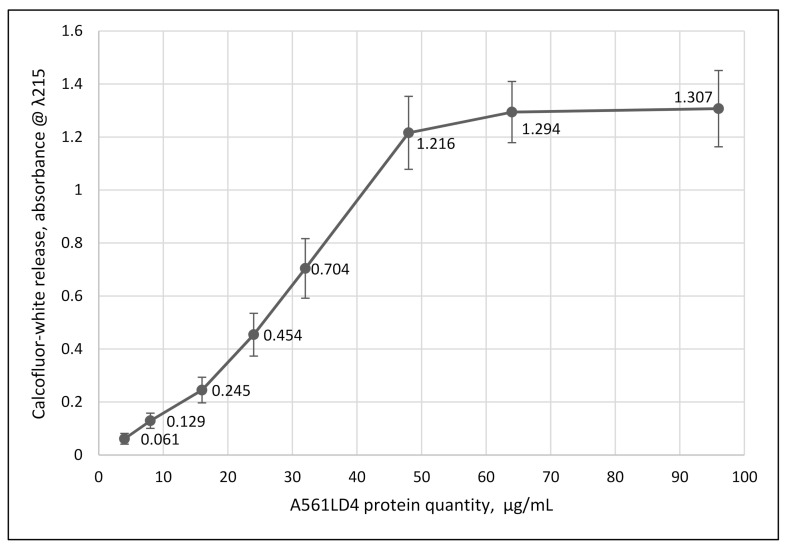
Specific activity of A561L^D4^ assayed by CFW release. Ghost chlorella cells (at a concentration of 5 × 10^7^/mL) stained with CFW were treated with the specified amount of recombinant A561L^D4^ for 1 h, then spun at 16,000× *g* for 15 min. The supernatant fraction was removed and CFW release was measured at λ = 215 nm. Results are plotted as mean ± standard deviation (*n* = 10), *p*-value 0.0001.

As reported in Figure 3, increasing concentrations of A561L^D4^ resulted in the release of CFW, demonstrating that the recombinant protein was active. Using the chlorophyll-release assay, A561L^D4^ was tested at different temperatures, pHs, and cation requirements. The protein was active from 10 °C to 60 °C, with an optimum near 37 °C, which is higher than the 25 °C optimum temperature for growing the host and the virus. The protein was active in pHs from 5.5 to 10.0 with an optimum between pH 7.0 and 8.0. The degradation activity was enhanced with Ca^2+^ and Mg^2+^ but it was also active without adding any cations probably due to leaking (residual) Ca^2+^ and/or Mg^2+^ cations from the live chlorella cells. Cell-wall degrading activity was inhibited by Mn^2+^, Cu^2+^, Co^2+^, Fe^2+^, Zn^2+^, Ni^2+^, EDTA and EGTA. The activity was destroyed by boiling or prior protease treatment.

### 3.4. Wall Degradation by A561L^D4^

**Figure 4:**
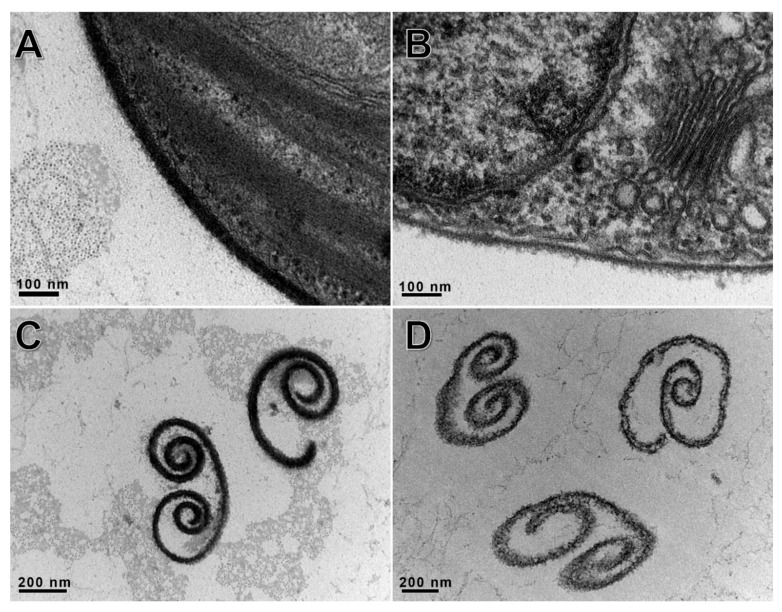
Degradation of *C. variabilis* NC64A cell walls after 10 min of digestion with 250 µg/mL of A561L^D4^ visualized by TEM. (**A**,**C**) were treated with the recombinant protein, (**B**,**D**) were controls. Top panels (**A**,**B**) represent live cells and bottom panels (**C**,**D**) cell wall fragments.

Exposure of live and ghost cells of *C. variabilis* NC64A to A561L^D4^ was examined by transmission electron microscopy (TEM). The walls were partially degraded by 10 min after addition of A561L^D4^ (Figure 4). This was confirmed using the chlorophyll-release assay and CFW-release assay.

**Figure 5:**
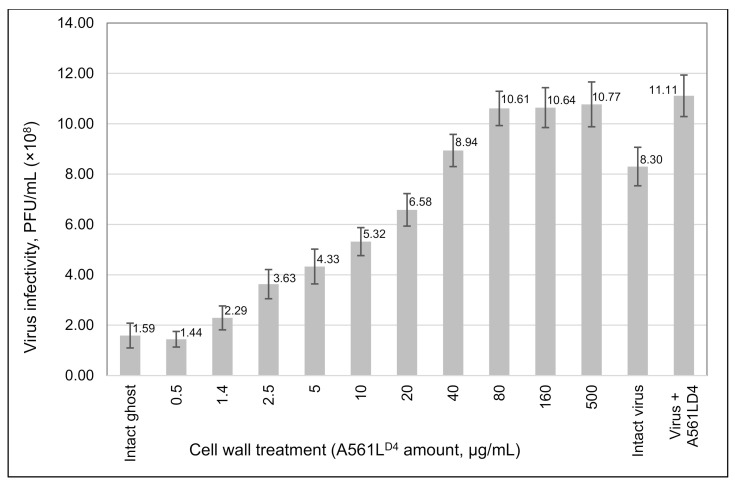
Effect of A561L^D4^ treatment of *C. variabilis* ghost cells on PBCV-1 virus receptors. Ghost cells (10^8^ cells/mL) in TB with 10 mM CaCl_2_ were treated with various amounts of A561L^D4^ for 24 h. After incubating the ghost cells, the reactions were centrifuged for 15 min at 16,000× *g*, (the supernatant fractions were collected for the experiments in Figure 6), and the pelleted cells were washed with TB one time and then re-suspended in 200 µL of TB with 10 mM CaCl_2_. PBCV-1 virus was added to the treated ghost cells at a MOI of 10 and incubated for 1 h at room temperature. Serial dilutions of the reaction were made and virus infectivity was plaque assayed. Results are plotted as mean ± standard deviation (*n* = 10), *p*-value 0.0001.

We hypothesized that A561L^D4^ treatment of isolated cell walls resulted in (i) the inactivation of the PBCV-1 receptor, and/or (ii) blocking the ability of the virus to adsorb to the cell. To test these two hypotheses, we isolated *C. variabilis* NC64A ghost cells (5–6 × 10^7^ cells/mL), incubated them with different concentrations of A561L^D4^ for various times, and collected the resulting residual cell wall material by centrifugation. The pellet fraction was re-suspended in TB and tested for its ability to interfere with PBCV-1 infectivity by incubating the fraction with virus, then evaluating by the plaque assay. As reported in Figure 5, increasing protein concentration and incubation time led to a decrease in the ability of the A561L^D4^-treated ghost cells to bind PBCV-1. The first conclusion from this experiment is that the host cell wall receptor for the virus was inactivated by exposure to A561L^D4^; however, it is also possible that A561L^D4^ was blocking the virus receptor such that the virus was unable to bind to the ghost cell.

**Figure 6:**
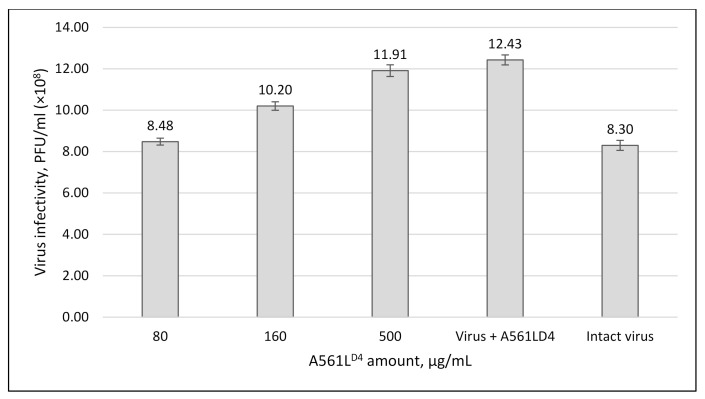
Effect of the soluble product resulting from A561L^D4^ treatment of *C. variabilis* NC64A ghost cells on PBCV-1 virus infectivity to determine if A561L^D4^ treatment resulted in the release of intact host receptor. Supernatant fractions collected from the experiment in Figure 5 were collected and PBCV-1 virus was added to the collected supernatant fraction at a concentration of 10^9^ PFU/mL and incubated for ~1 h at room temperature. Serial dilutions of the supernatant fractions were made and virus infectivity was plaque assayed. Clearly, the host receptor was not released intact by A561LD4. Note, however, that exposure of purified PBCV-1 to A561L^D4^ resulted in an ~50% increase in the specific infectivity of PBCV-1. Results are plotted as mean ± standard deviation (*n* = 10), *p*-value 0.0001.

The supernatant fraction was collected in the above experiment and used to determine if A561L^D4^-treated ghost cells resulted in the release of a soluble factor that prevented PBCV-1 adsorbing to the live cells. If this occurred, the soluble factor could be the intact viral binding receptor, which would prevent the virus from binding to the algal cell to initiate infection in the plaque assay. However, as reported in Figure 6, exposure of PBCV-1 to the supernatant fraction prior to the plaque assay did not result in a decrease in PBCV-1 titers. Therefore, the A561L^D4^-treated ghost cell wall did not release a soluble factor capable of blocking virus adsorption, thereby excluding the presence of an intact cell-wall receptor in the soluble fraction. Currently, the nature of the chlorovirus receptor is unknown, but treating ghost cell walls with standard proteases does not interfere with virus binding; however, certain alginate lyase preparations reduce virus binding, suggesting that the receptor is composed of a type of carbohydrate [24].

### 3.5. A561L^D4^ Treatment Increased PBCV-1 Infectivity

As a control in the previous experiment, highly purified PBCV-1 was incubated with A561L^D4^ and we were surprised to discover that exposure of PBCV-1 to A561L^D4^ resulted in an almost 50% increase in the specific infectivity of PBCV-1 compared to untreated, purified PBCV-1 (Figure 6). It is worth noting that we repeatedly observed similar increases in virus specific infectivity after treatment with vLysin [25].

We have previously reported that about 25 to 30% of highly purified PBCV-1 virions form plaques [29]. We suspect that this unexpected finding occurred because the purified viruses contained residual host receptor material attached to their spike structures. This putative residual receptor material was then digested by A561L^D4^, leading to an increase in infectious viruses.

### 3.6. Immunological Detection of the A561L Protein in the Virion

We examined the possibility that A561L might be post-translationally cleaved into two or more peptides, including one peptide consisting solely of A561L^D4^ in the virus particle. However, two experiments ruled out this possibility. First, the mass spectrometry proteomic data suggested that the entire protein was present in the virion [16].

**Figure 7:**
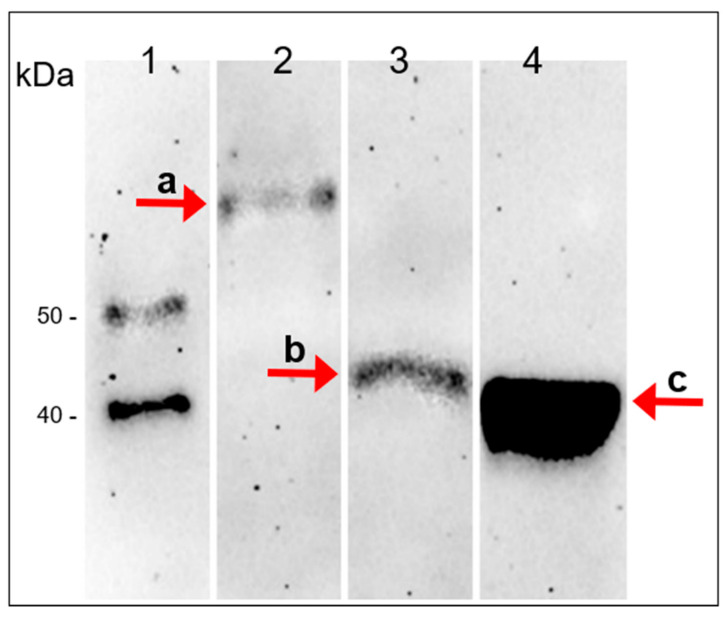
Presence of A561L protein in the PBCV-1 virion. Western blot of PBCV-1 proteins against anti A561L^D4^ antibody (AB) produced in mice. (**1**). protein ladder; (**2**). untreated virus; (**3**). virus treated with 5 M LiCl (vLysin); (**4**). recombinant A561L^D4^. Band **a** full length A561L protein ~72 kDa; band **b** processed fragment of A561L ~44 kDa (vLysin activity); band **c** recombinant A561L^D4^ protein ~42 kDa (the A561LD^4^ domain is ~27 kDa plus His-Patch thioredoxin, V5 epitope tag and 6xHis tags).

Second, a mouse polyclonal antibody to A561L^D4^ was produced and used in a western blot analysis of total PBCV-1 protein. The blot indicated that the antibody reacted with a protein of about 72 kD, the expected size of A561L (Figure 7, band **a**). However, unexpectedly LiCl extracted vLysin led to the formation of a 42 kDa protein (Figure 7, band **b**). This unexpected finding suggests that the A561L protein might have a protease sensitive region that leads to the release of a C-terminal end peptide approximately encompassing both domain three (A561^D3^) and domain four (A561L^D4^). At this point we do not know if this cleavage actually occurs during virus infection. However, it is worth noting that domain two (A561^D2^) resembles a peptidase S8 domain-containing protein from *Tetradesmus obliquus* with an E-value of 9.3 × 10^−8^. This putative peptidase domain might serve as a cis-acting protease and cleave itself under appropriate conditions.

### 3.7. A561L Is Located Inside the PBCV-1 Virion

Two experiments indicate that A561L is probably located inside the PBCV-1 particle rather than on the surface. First, treatment of purified viruses with proteinase K had no effect on virus infectivity as judged by the plaque assay [16]. Second, addition of anti-A561L^D4^ polyclonal antibody to PBCV-1 significantly decreased the virus’s ability to digest the host’s cell wall (down to 22% compare to control) but did not terminate it completely as judged by the chlorophyll release assay (data not shown). Therefore, we suspect that the protein is located either inside the PBCV-1 spike structure or in the cavity that is located between the virus internal membrane and the base of the spike (see Figure 3a in Zhang et al. [9]).

**Figure 8:**
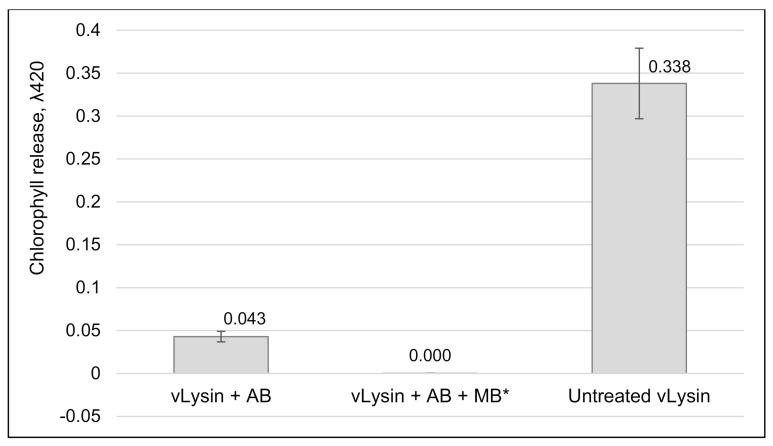
Effect of anti A561L^D4^ AB on vLysin activity. PBCV-1 isolated vLysin was treated with anti A561L^D4^ antibody (AB) produced in mice (800-fold dilution) for 1 h. After the incubation, anti-mouse Ig MB were added to the reaction and the supernatant fraction was separated from the magnetic beads following the manufacturer’s instructions. Antibody treated vLysin was mixed with NC64A cells and incubated for 1 h. The treated cells were subject to 1% SDS, spun down, the supernatant fraction was removed and chlorophyll release was measured at λ = 420. Untreated PBCV-1 vLysin was used as a positive control. NC64A cells treated with pre-bleed serum served as a negative control. Results are plotted as mean ± standard deviation (*n* = 4), *p*-value 0.0001. (*) A mean of standard deviation for vLysin + AB + MB was 0.0002.

The anti-A561L^D4^ antiserum was also used to determine if vLysin wall-degrading activity was the only cell wall degrading activity packaged in the virion. As reported in Figure 8, addition of the anti-A561L^D4^ antiserum at 800-fold dilutions to vLysin blocked chlorophyll release from *C. variabilis* NC64A. This complete inhibition of activity indicates that A561L^D4^ is probably the only cell wall degrading activity in the virion.

### 3.8. A561L^D4^ Homologs Are Common among the Chloroviruses

**Figure 9:**
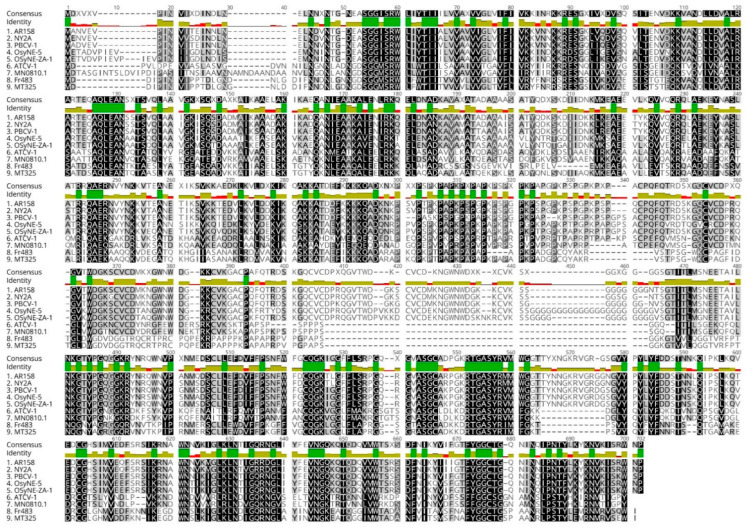
ClustalW alignment of A561L homologs among representatives of four clades of chloroviruses. The similarity of amino acids in the sequence alignment is shown as follows: black being identical, different shades of gray (different levels of conservation) and white not conserved. On the top ribbon: green color denotes identical amino acids. Other shades of amino acids indicate a level of conservation from olive color as being the most conserved to red color not conserved. AR-158, NY-2A along with PBCV-1 are NC64A viruses; OsyNE-5 and OsyNE-ZA-1 infect Syngen strain; ATCV-1 and MN0810.1 are SAG viruses; Fr-483 and MT325 are Pbi viruses. A561L^D1^ domain ends at aa 93, domain A561L^D2^ stretches from aa 94 to aa 338, domain A561L^D3^ runs from aa 339 to aa 456 and domain A561L^D4^ starts at 457 aa.

If A561L is important for virus genome entry into its host, it would be expected to be present in all of the chloroviruses. Therefore, annotated genomes of 52 chloroviruses were searched for homologs to the *a561l* gene. The investigation revealed that all 52 sequenced viruses encoded an A561L-like protein with a lyase domain (i.e., D4 like) located in the C-terminus of the protein. A clustalW alignment of A561L homologs from eight chloroviruses plus PBCV-1, consisting of two representatives from each of the four subtypes of chloroviruses is reported in Figure 9. Overall, there was ~32% amino acid identity and 48% amino acid similarity in the A561L homologs among the 52 viruses. Therefore, A561L was considered a core chlorovirus protein.

### 3.9. Phylogenetic Analysis of A561L

**Figure 10:**
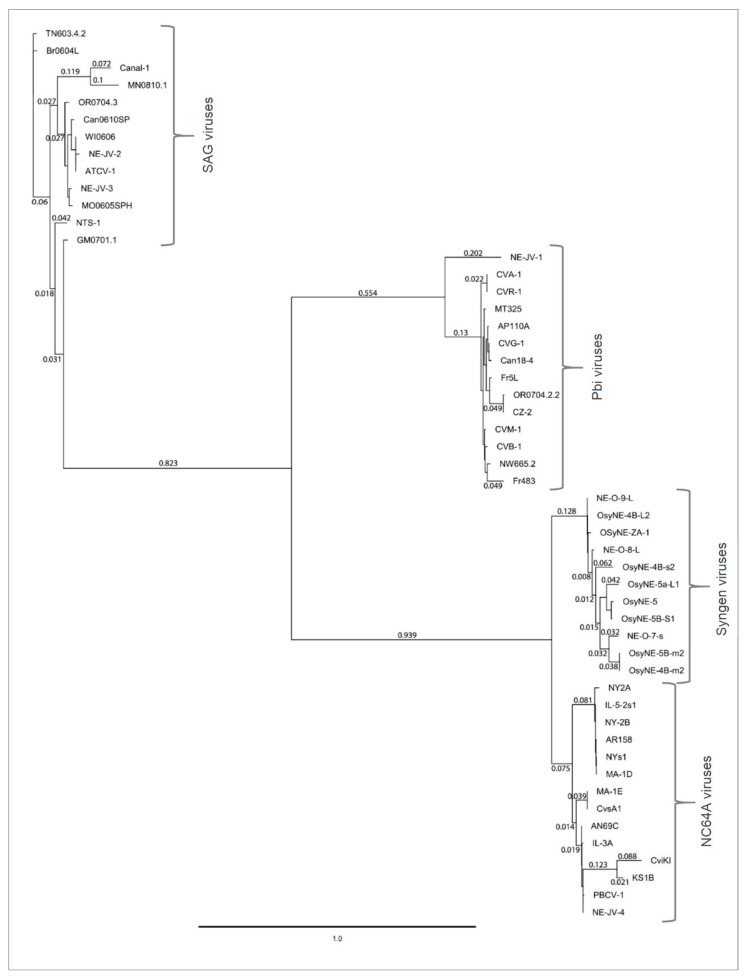
Phylogeny of A561L protein from 52 chloroviruses. Phylogenetic tree was constructed with Geneious plugin program PhyML 3.3.20180621 (Maximum likelihood) [33] using the default settings. The bar indicates the number of substitutions per site and branch length shows dissimilarity between strains.

Homologs of A561L protein coded by the 52 chloroviruses were aligned and a phylogenetic tree was constructed using PhyML, a maximum likelihood method (Figure 10). Alignment results showed that the most conserved A561L domains are domain 2 (A561L^D2^) and domain 4 (A561L^D4^). The A561L proteins clustered into the four clades of chloroviruses that are based on their algal hosts.

The percent amino acid identity between the clades representing the different host/virus groups was: 27.9–36.0% identity between NC64A—SAG clusters, 32.2–38.4% identity between NC64A—Pbi clusters and 36.9–41.7% identity between Pbi—SAG clusters. The range of protein sequence identity within-clades was 65.0–100%, 73.4–100% and 71.1–100% identity for NC64A, SAG and Pbi viruses, respectively.

### 3.10. A561L^D4^ Degrades the Cell Wall of Several Chlorella Species

To determine the specificity of A561L^D4^ activity, the protein was tested on 27 green algal isolates (Table 1). However, it was first necessary to establish that the algal isolates were resistant to 1% SDS as judged by the chlorophyll-release assay (Figure 2A). The results (not shown) demonstrated that all algal isolates were resistant to 1% SDS. Therefore, each of the 27 algal isolates (at a concentration of 2 × 10^7^ cells/mL) were exposed to 100 µg/mL of A561L^D4^ for 1 h and examined for chlorophyll release. The range of the released chlorophyll cannot be directly compared between algal isolates due to the innate differences in the strain physiology and cell chlorophyll content. The experiment showed that A561L^D4^ was active on the other three chlorovirus hosts, *C. variabilis* Syngen 2-3, *C. heliozoae* and *M. conductrix*, as well as eight other green algal isolates (Table 1).

The green alga, *Chlamydomonas reinhardtii*, which has a very different cell wall composition [40] than *Chlorella* (e.g., [41]), was sensitive to all the detergents that we tested and so it could not be assayed for sensitivity to A561L^D4^ using the chlorophyll release assay.

## 4. Conclusions

Unexpectedly, none of the five previously characterized virus-encoded polysaccharide degrading enzymes are packaged in the PBCV-1 virion; consequently, they were eliminated as candidates for cell wall penetration during the entry phase of PBCV-1 infection of its host, *C. variabilis* NC64A. However, A561L is a virus-encoded protein that is found in the virion, and we provide evidence that it is involved in degrading the host cell wall during viral entry. The A561L protein has four putative domains; the C-terminal domain consisting of 237 amino acids has the cell wall degrading activity, which we referred to as A561L^D4^. A561L^D4^ was expressed as a recombinant soluble protein and shown to partially degrade *C. variabilis* NC64A cell walls. All 52 chloroviruses that have been sequenced, infecting four different hosts, had an *a561l* homolog and the cell wall of all four chlorovirus hosts, as well as several non-host *Chlorella* spp., were partially degraded by A561L^D4^. Thus, A561L^D4^ was not cell-type-specific. Finally, we discovered that exposure of highly purified PBCV-1 virions to A561L^D4^ increased the specific infectivity of PBCV-1 from about 25–30% of the virus particles forming plaques to about 50% of the particles forming plaques. We attribute this increase to removal of residual putative host receptor material that was attached to newly replicated viruses in the cell lysates.

## Figures and Tables

**Table 1:** Sensitivity of different green algae to recombinant A561L^D4^ overexpressed protein.

Species	Strain Designation	Chlorophyll Release, λ = 420 ^#^
*Auxenochlorella protothecoides*	stock 29	0.840
*Chlorella heliozoae*	SAG 3.83	0.570
*Chlorella miniata*	UTEX 490	0.198
*Chlorella sorokiniana*	UTEX 246	0.335
*Chlorella sorokiniana*	UTEX 1810	0.294
*Chlorella sorokiniana*	UTEX 1230	0
*Chlorella sorokiniana*	CS01 China	0
***Chlorella variabilis***	**NC64A**	**0.267**
*Chlorella variabilis*	NIES 2540	0.236
*Chlorella variabilis*	NIES 2541	0.368
*Chlorella variabilis*	Syngen 2-3	0.229
*Chlorella vulgaris*	UTEX 395	0.687
*Chlorella* sp.	UTEX BSN 069	0.681
*Chlorella* sp.	UTEX BSN069	0.507
*Chloroidium saccharophilum*	UTEX 247	0
*Coccomyxa subellipsoidea*	C169	0
*Micractinium conductrix*	Pbi	0.376
*Parachlorella kessleri*	UTEX 2228	0
*Scenedesmus dimorphus*	UTEX 417	0.392
*Scenedesmus dimorphus*	UTEX 1237	0
*Scenedesmus obliquus*	UTEX B2630	0
*Scenedesmus obliquus*	UTEX 393	0
*Scenedesmus obliquus*	UTEX 1450	0
*Scenedesmus parisiensis*	UTEX 1585	0
*Scenedesmus* sp	UTEX 1590	0
*Scenedesmus* sp	UTEX 1589	0
*Scenedesmus* sp	UTEX 2193	0
*Scenedesmus* sp	UTEX 1591	0

^#^ Green algae (at a concentration of 2 × 10^7^ cells/mL) were treated with 100 µg/mL A561L^D4^ protein for 1 h, then treated with 1% SDS, incubated for 1 min; cells were spun down for 15 min at 16,000× *g*; the supernatant fraction was removed and chlorophyll release was measured at λ = 420. Control cells were treated with TB.

## Supplementary Materials

The following are available online at https://www.mdpi.com/article/10.3390/v13050782/s1, Table S1: Attributes and sequences of chlorovirus encoded homologs of *A561L* gene.

## Appendix A

**Table A1:** Summary of Phyre2 top four templates for protein structure prediction.

Template, PDB	Template Information	Confidence, %	% i.d.	% of Coverage
c5gmtB	crystal structure of the marine pl-14 alginate lyase from *Aplysia kurodai*	100	23	85
c6kcvB	structure of alginate lyase from *Chitinophaga* sp. MD30 aly36b mutant k143a/y185a in complex with alginate tetrasaccharide	100	23	87
c3im0A	crystal structure of chlorella virus Val-1	100	30	87
c2zzjA	crystal structure of endo-beta-1,4-glucuronan lyase from fungus *Trichoderma reesei*	99.7	16	79
